# Neural and Behavioral Correlates of Attentional Inhibition Training and Perceptual Discrimination Training in a Visual Flanker Task

**DOI:** 10.3389/fnhum.2018.00191

**Published:** 2018-05-23

**Authors:** Robert D. Melara, Shalini Singh, Denise A. Hien

**Affiliations:** ^1^Department of Psychology, The City College of New York, The City University of New York, North Academic Center, New York, NY, United States; ^2^Derner School of Psychology, Adelphi University, Garden City, NY, United States

**Keywords:** rejection positivity, inhibition training, attention skill, flanker task, dipole source

## Abstract

Two groups of healthy young adults were exposed to 3 weeks of cognitive training in a modified version of the visual flanker task, one group trained to discriminate the target (discrimination training) and the other group to ignore the flankers (inhibition training). Inhibition training, but not discrimination training, led to significant reductions in both Garner interference, indicating improved selective attention, and in Stroop interference, indicating more efficient resolution of stimulus conflict. The behavioral gains from training were greatest in participants who showed the poorest selective attention at pretest. Electrophysiological recordings revealed that inhibition training increased the magnitude of Rejection Positivity (RP) to incongruent distractors, an event-related potential (ERP) component associated with inhibitory control. Source modeling of RP uncovered a dipole in the medial frontal gyrus for those participants receiving inhibition training, but in the cingulate gyrus for those participants receiving discrimination training. Results suggest that inhibitory control is plastic; inhibition training improves conflict resolution, particularly in individuals with poor attention skills.

## Introduction

How does cognitive experience shape a viewer’s ability to control visual attention? Recent research suggests that rigorous training to more efficiently employ certain executive functions, including selective attention and working memory, can yield long-lasting improvements to behavioral performance on cognitive tasks (Thorell et al., 2009; Diamond and Lee, 2011; Jaeggi et al., 2014), including limited transfer to untrained tasks (Garner et al., 2015), while also altering neural activity (Rueda et al., 2012; Tang and Posner, 2009; Millner et al., 2012), especially among those with poor attentional abilities (Diamond and Lee, 2011). Cognitive training also has proven clinical benefits, alleviating symptoms of depression, anxiety, and Attention Deficit Hyperactivity Disorder (Willcutt et al., 2005; Eldar and Bar-Haim, 2010; Sari et al., 2016), and improving emotion regulation (Rueda et al., 2012). Yet training aimed at better inhibiting distraction has been mostly relegated to the auditory modality, and there only in situations free of stimulus conflict. The purpose of the present study was to explore how specific forms of cognitive training in visual attention may differentially affect behavioral performance and neural functioning in tasks requiring the resolution of stimulus conflict.

### Types of Training

Attention has historically been viewed as a cognitive process designed primarily to enhance task-relevant information (Egner and Hirsch, 2005; Aron, 2007). However, a growing body of evidence points to two dissociable attentional mechanisms operating concurrently whenever observers perform executive control tasks: the activation of task-relevant information and the inhibition of task-irrelevant information (Couperus and Mangun, 2010; Liu et al., 2014; Noonan et al., 2016).

Recent evidence suggests that distinct forms of cognitive experience may differentially influence these two mechanisms. Melara et al. (2002), for example, assigned participants randomly to 3 weeks of auditory training aimed at either boosting the efficiency of target processing (practice at auditory frequency discrimination) or suppressing the disruptive impact of salient distractors (practice at ignoring irrelevant tones). The effects of training were assessed in a dual-channel auditory selective attention task performed a week before and a week after the training regimen. The researchers found that participants could reduce attentional interference after distractor inhibition training, but not after target discrimination training, a skill participants were then able to transfer from trained to untrained auditory frequencies. Measurement of event-related potentials (ERPs) revealed that inhibition training increased the amplitude of a slow wave to the distractors, known as Rejection Positivity or RP (i.e., a slow wave ERP component that appears approximately 200 ms after distractor onset and can last 400 ms or more thereafter; Chen and Melara, 2014), and reduced the amplitude of the P3 component to the distractors, both of which were strongly associated with behavioral performance, suggesting an inhibition-specific neural basis to the training effect (see also Andersen and Müller, 2010; Itthipuripat et al., 2013). Melara et al. (2012) discovered that these gains in performance were retained even 4 weeks after training, with enhancement in RP actually growing during the follow-up period, with no further training (see also Tallus et al., 2015).

During selective attention performance, inhibitory control can dampen distractor activation; the RP and P3 ERP components index the course and extent of dampening (Melara et al., 2002; Münte et al., 2010; Mittag et al., 2013). The P3 wave, for example, provides an electrophysiological gauge of the salience of stimuli held in working memory (Karis et al., 1984; Donchin and Coles, 1988). To the degree that participants learn to inhibit distractors, one would expect a decrease in distractor salience in working memory (Wilken and Ma, 2004), measured by weakened P3 amplitude to distractors, as Melara et al. (2002) found. Yet extant comparisons pitting discrimination to inhibition training have been limited to the auditory modality. In the current study, we explored the effect of discrimination and inhibition training on RP and P3 amplitude during visual selective attention.

The effects of executive control on RP and P3 components suggest activation to specific neural regions. In an examination of scalp topographies, for example, Bidet-Caulet et al. (2010) identified the center-of-mass of RP at frontal electrode sites, suggesting an inhibitory control signal sourced in frontal lobe. Moreover, in certain models of attention (Botvinick et al., 2001; Kerns et al., 2004) control adjustments imposed by the prefrontal cortex (PFC), perhaps reflected in RP amplitude, serve to suppress distraction during successful selective attention. Yet the specific neural locus of training on RP is unknown. In the current study, we sought to localize the effects of training on executive control processes using dipole source analysis.

### Separate Measurement of Target and Distractor Processing Allows Differentiation Between Inhibition and Discrimination Training

Melara et al. (2002) and Tong et al. (2009) used training to explore participants’ ability to avoid distraction. However, another essential function of attention is to resolve conflict. Millner et al. (2012) trained participants on one conflict task (Simon task) to measure transfer to another conflict task (Eriksen flanker task). Training sped responses on trials when flankers were incongruent with targets and, importantly, attenuated the magnitude of the N2 ERP component on these trials. The N2 component is associated with conflict detection and resolution, with neural sources in the dorsal anterior cingulate cortex (Mathalon et al., 2003; Silton et al., 2010). Thus, the results of Millner et al. (2012) suggest that cognitive training can improve the speed and efficiency of conflict resolution (see also Garner et al., 2015). Unfortunately, the study was limited by the absence of a control group and the simultaneous presentation of targets and flankers, which prevented the authors from disentangling the effects of training on target processing versus distractor processing.

In the current study, we developed a modified version of the visual flanker task (Eriksen and Eriksen, 1974) that enabled us on each trial to take separate electrophysiological recordings of targets and distractors. We thus were able to measure N2 amplitude specifically to targets before versus after training. We asked whether inhibition and discrimination training differentially affect target processing. In this way, we were able to perform a more granular analysis than hitherto of the processes involved in the detection and resolution of stimulus conflict.

### Individual Differences in Attention Skill Moderates Training Outcomes

Evidence suggests that the impact of cognitive training may depend in part on an individual’s initial level of executive function. Participants with high scores on tests of working memory perform better than those with low scores on tasks of selective attention (Engle et al., 1999; Long and Prat, 2002; Heitz and Engle, 2007). Gains in attention training are greatest for individuals with the poorest executive functions (Diamond and Lee, 2011; Millner et al., 2012). And, individual differences in working memory influence how participants use attention when performing cognitive tasks (Buschkuehl et al., 2012; Gulbinaite et al., 2014). In the current study we asked whether individuals high versus low in selective attention skills benefit more from the two types of cognitive training.

### The Current Study

Here, we examined group and individual differences in behavior, ERP magnitude, and source localization during visual selective attention following 3 weeks of either target discrimination training or distractor inhibition training. Our specific hypotheses were: (1) Attention training would decrease the amplitude of both the N2 waves to incongruent targets, which gauges the ability to monitor and resolve conflict, as suggested by the research of Millner et al. (2012) (see also Garner et al., 2015), and the P3 waves to flankers, which gauges the ability to suppress distractors, as suggested by the research of Melara et al. (2002); (2) Participants with the poorest premorbid attentional control would show the greatest gains from attention training, in line with previous studies on attention skill (Diamond and Lee, 2011; Millner et al., 2012); and (3) Inhibition training would enhance the RP wave to distractors, in keeping with results of previous studies in the auditory modality comparing discrimination and inhibition training (Melara et al., 2002, 2012), perhaps through increased activation of PFC, as revealed in dipole source analysis.

## Materials and Methods

### Participants

Thirty-six participants (25 females, average age = 20.4 years), recruited from The City College of New York, were given course credit for participation in the study. The nature of the procedures was explained fully, and informed consent was obtained from each participant; the Institutional Review Board of The City University of New York approved the protocol. All participants had normal or corrected-to-normal vision with no history of neurological disorder (self report). Participants were assigned randomly to either target discrimination training (18 participants, 13 female) or distractor inhibition training (18 participants, 12 female).

### Stimulus, Apparatus, and Procedure

**FIGURE 1:**
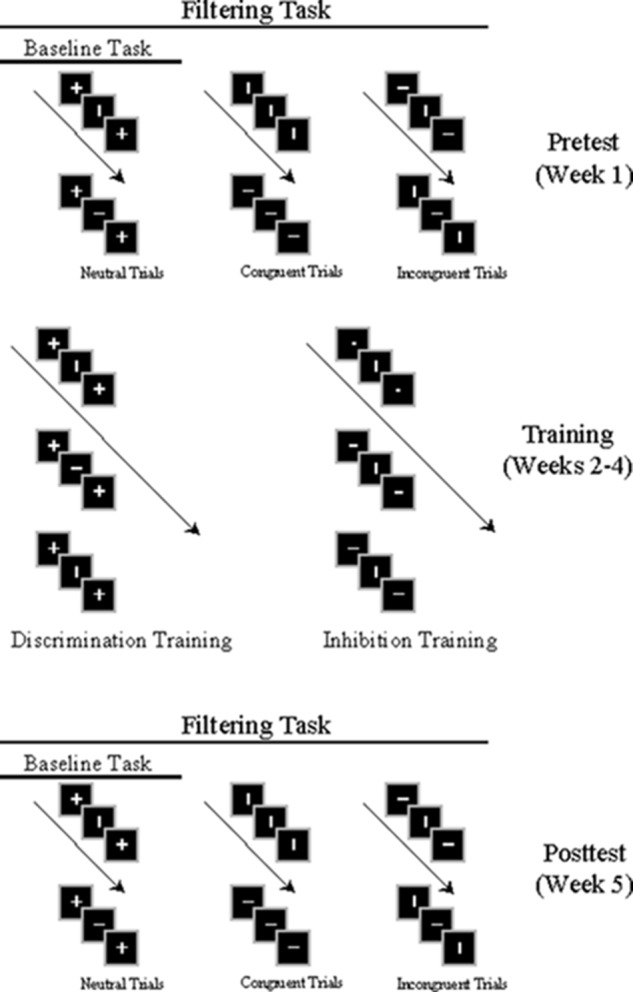
Depiction of pretest (Week 1), training (Weeks 2–4), and posttest (Week 5) in temporal flanker task. Pretest and posttest include baseline and filtering tasks; filtering tasks comprise neutral, congruent and incongruent trials. Participants engaged in either discrimination or inhibition training.

The study lasted 5 weeks. Each participant was tested in a pretest session (1st week) and a posttest session (5th week) occurring at the same time of day each week in an electrically and acoustically shielded Industrial Acoustics Company (New York) chamber while EEG was recorded (see below for details). Each test session contained 20 blocks of 100 experimental trials (and one or more blocks of practice trials) in a modified version of the visual flanker task called the temporal flanker paradigm. Stimuli were created in Presentation^®^ (Neurobehavioral Systems) and appeared to participants as they sat in a comfortable chair at a distance of 60 cm from a Dell Model P1130 RGB computer monitor with a refresh rate of 75 Hz. Each trial consisted of a fixation square (0.67°) followed by three stimulus displays presented sequentially: (1) First Flanker, (2) Target, and (3) Second Flanker (see **Figure 1**). Each display appeared for 150 ms separated by an inter-stimulus interval varying between 153 and 390 ms in random distribution. The first and second flankers were identical on each trial: a vertical line, a horizontal line, or a cross. Line stimuli, subtending a visual angle of 0.47°, appeared in gray on a black background. On each trial of each task participants were asked to respond by mouse key as quickly and accurately as possible to the orientation of the target line, ignoring the flanker lines. Assignment of line orientation to response keys was counterbalanced across participants.

Test sessions were divided into 10 baseline and 10 filtering conditions (Garner, 1974) performed as a set, with task order balanced across participants. In each baseline task, flankers on each trial were neutral crosses. Baseline tasks performed below 80% accuracy were repeated immediately. In each filtering task, the three types of flankers (vertical line, horizontal line, or cross) appeared randomly as a pair on each trial: On 15% of trials (15 of 100) target and flanker lines matched in orientation (congruent trials), on 15% they mismatched (incongruent trials), and on 70% (70 of 100) flankers were crosses (neutral trials). Flankers always matched each other on each trial. Participants were given short breaks throughout testing. The entire experiment, including EEG preparation, lasted approximately 3 h.

Three training sessions, each involving 24 blocks of 100 trials, were held during the second, third, and fourth weeks. During discrimination training only baseline tasks (neutral flankers, 0.57°) were employed. The purpose of discrimination training was to provide participants with practice in identifying targets in the absence of distraction (see Melara et al., 2002). During inhibition training only filtering tasks (congruent, incongruent, and neutral flankers) were employed. The purpose of inhibition training was to expose the participants to progressively stronger degrees of the irrelevant signal to improve their ability to suppress distracting events while resolving conflict. Hence, the majority of trials in inhibition training were incongruent (60%; congruent = 20%; neutral = 20%). Participants performed eight sets of inhibitory training during each training week, with the signal-to-noise ratio progressively reduced by increasing the perceptibility of flankers across the three filtering tasks in each set: low salience filtering (target/flanker ratio = 0.47°/0.19° = 2.47), medium salience filtering (target/flanker ratio = 0.47°/0.29° = 1.62), and high salience filtering (target/flanker ratio = 0.47°/0.38° = 1.24). Training for each participant occurred at the same time of day each week as the pretest and posttest.

### Data Recording and Analysis

Participants performed the visual flanker task in baseline (only neutral flankers) and filtering (congruent, incongruent, and neutral flankers) conditions at both pretest and posttest while behavioral and electrophysiological measures were made, allowing us to derive three separate indices of selective attention: Garner interference (difference between baseline and filtering conditions), Stroop congruity (difference between congruent and incongruent trials), and distraction recovery (difference to neutral trials before versus after conflict). Response accuracy (percent correct) and reaction times (RTs) to correct response trials were averaged for each participant in each condition. We performed mixed model analyses of variances (ANOVAs) on behavioral data using Statistica^®^ software, with Training Group (two levels: Discrimination Training and Inhibition Training) and Attention Skill (two levels: good attenders and poor attenders) as the between-subjects factors, and Task (two levels: baseline and filtering) and Test (two levels: pretest and posttest) as within-subject factors. To meet assumptions in ANOVA, accuracy analyses were carried out on arcsine-transformed data (Winer et al., 1991). In analyses of flanker effects in filtering tasks, Congruity (three levels: congruent, incongruent, and neutral) and Recovery (two levels: before conflict and after conflict) replaced Task in ANOVA. To define Skill operationally, participants were divided into good or poor attenders using a median split of the magnitude of Garner interference in RT at pretest (Filtering RT minus Baseline RT) in task analyses or Congruity effect in RT at pretest (Incongruent RT minus Congruent RT) in flanker analyses. To guard against violations of the sphericity assumption with repeated-measures data, all main effects and interactions reported as significant were reliable after Greenhouse-Geisser correction (Greenhouse and Geisser, 1959).

During pretest and posttest sessions (but not training sessions) continuous recordings of the EEG were collected at a sampling rate of 512 Hz using a BioSemi Active-Two system in a high-density (160 electrodes) montage arranged in an elastic cap. Blinks and other eye movements were monitored by electrooculogram (EOG) from two electrode montages, one on the infra- and supra-orbital ridges of the right eye (VEOG), the other on the outer canthi of each eye (HEOG). Trials containing mastoid activity exceeding 100 μV were rejected. Trials contaminated by blinks, eye movements, or other movement artifacts were defined as *z*-values on the VEOG, HEOG, and lowermost scalp channels exceeding 4.5 in a frequency band between 1 and 140 Hz; artifact trials were removed automatically using a Matlab routine (Fieldtrip; Oostenveld et al., 2011).

**FIGURE 2:**
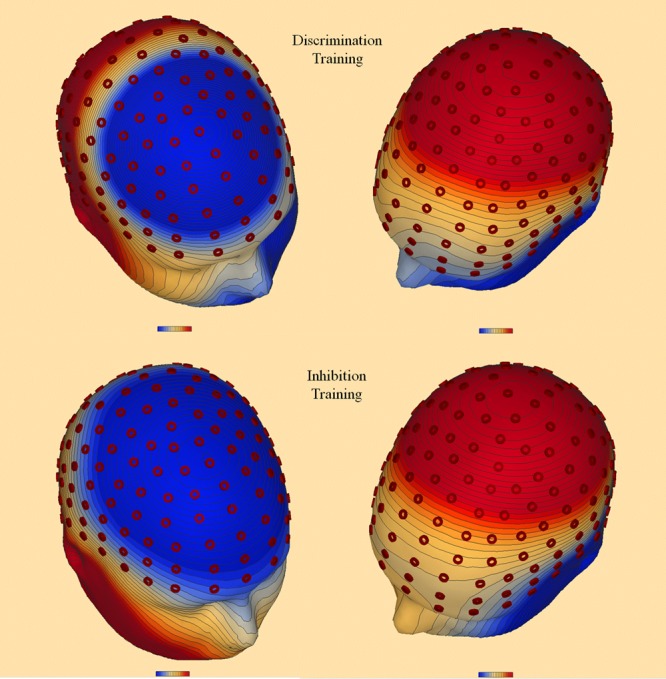
Topographic voltage maps to targets at pretest during N2 (200–400 ms after stimulus onset; Left) and P3 (400–900 ms after stimulus onset; Right) time epochs, separately for the discrimination group (Top) and the inhibition group (Bottom). Center-of-mass for N2 was localized over central electrode sites and for P3 over parietal electrode sites.

Event-related potentials were restricted to trials involving a correct behavioral response to targets. Sweep time to each stimulus (target and initial flanker) was 1200 ms, including a 200 ms pre-stimulus (re: target or flanker) baseline; signal-averaged waveforms were referenced to linked mastoids band-pass filtered between 0.1 and 30 Hz. ERP components were measured to the target (N2 and P3 components) and the initial flanker (RP and P3 components), separately for congruent, incongruent, and neutral stimuli. Target N2 amplitude was defined as the peak negative amplitude to the target 200–400 ms after stimulus onset and was measured over nine averaged Biosemi central scalp locations: A1, C1, B1, A2, E1, D1, E24, A3, and B2 (Mathalon et al., 2003). P3 amplitude was defined as the peak positive amplitude 400–900 ms after stimulus onset, separately measured to target and distractor stimuli over nine averaged parietal electrode locations: A2, A3, A4, A19, A20, E24, B2, A5, and A32 (Donchin and Coles, 1988). Topographic voltage maps within the N2 and P3 time epochs confirmed in each group a center-of-mass over central and parietal electrode sites, respectively (see **Figure 2**). Distractor RP was defined as the average voltage to the initial flanker 600–1000 ms after stimulus onset and measured over seven frontal electrode locations: D7, D8, C18, C31, C32, D11, and D22 (Bidet-Caulet et al., 2010). Average RT to the target in the current study (approximately 500 ms) occurred well within the distractor RP epoch (i.e., 150 ms [duration of flanker] + 271 ms [average ISI between flanker and target] + 500 ms [average filtering RT to target) = 921 ms), providing a methodological basis for our theoretical view of RP as an inhibitory process contributing to target selection and decision making processes. ANOVAs of ERP amplitudes mimicked behavioral analyses, with Training Group (two levels) and Attention Skill (two levels) as between-subjects factors, and Test (two levels) and Congruity (two levels: congruent and incongruent) as within-subjects factors. To evaluate possible moderating effects on ERP amplitude of group differences in RT at pretest, we conducted a series of ANCOVAs using the difference at pretest in RTs between congruent and incongruent stimuli as a covariate in all ERP analyses. All main effects and interactions reported as significant were reliable after Greenhouse-Geisser correction (Greenhouse and Geisser, 1959). To probe brain-behavior relationships in executive control, we performed linear regressions in the filtering task between the inhibitory control measure, RP, and the two behavioral measures, RT and accuracy.

We used high-density ERP recordings to perform discrete dipole source analysis of conflict trials in the filtering task to identify brain sources of discrimination and inhibition training. Source analysis (Scherg and von Cramon, 1986) was performed across 160 scalp locations on a three-shell (skin, skull, cerebrospinal fluid/ brain) spherical Finite Element Method head model using Brain Electrical Source Analysis (BESA) software, separately for each group after training. The conductivity ratio of brain to skull was 80. Skull and skin thickness at the upper head was 7 mm; bone conductivity was 0.0042. Radial bone conductivity was three times weaker than tangential bone conductivity. Two dipoles were fit without constraints on location or orientation. Additional dipoles did not significantly reduce residual variance. All dipoles are reported in Talairach coordinates.

## Results

### Behavioral Performance

**FIGURE 3:**
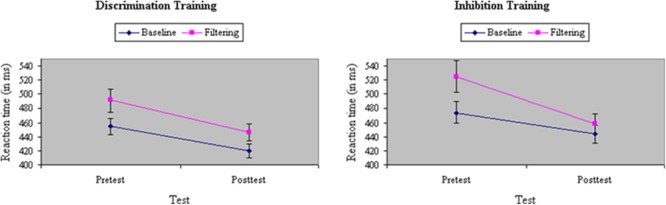
Reaction time (in ms) in baseline and filtering tasks at pretest and posttest in discrimination and inhibition training groups. Participants assigned to inhibition training showed a significantly greater reduction from pretest to posttest in Garner RT interference than participants assigned to discrimination training, yielding a significant Train × Test × Task interaction.

**FIGURE 4:**
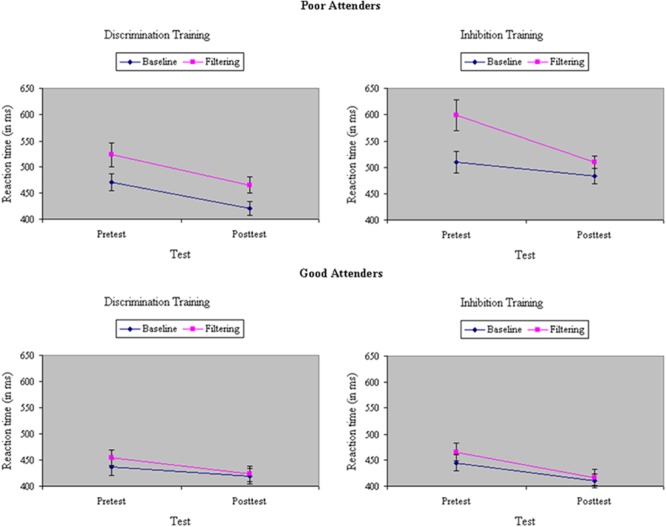
Reaction time (in ms) in baseline and filtering tasks at pretest and posttest in discrimination and inhibition training groups, separately by poor attenders (Top) and good attenders (Bottom). Reductions in Garner RT interference from inhibition training were restricted to participants showing relatively poor selective attention at pretest, yielding a significant Skill × Train × Test × Task interaction.

Average RT and accuracy in each condition at pretest and posttest appear in **Table 1**. The correlation between the two behavioral measures, computed across groups (discrimination training, inhibitory training), test sessions (pretest, posttest), and stimulus types in the filtering task (congruent, incongruent, neutral) was *r* = -0.84, suggesting that there was no trade off between speed and accuracy. An ANOVA of RTs yielded a main effect of Task, *F*(1,33) = 108.39, *p* < 0.001, MS_e_ = 365.69, η^2^= 0.11, with performance in the filtering task (483 ms) 33 ms slower on average than performance in the baseline task (450 ms), indicating a failure of selective attention to the random presentation of distractors. Participants assigned to inhibition training showed a slightly greater difference before training in performance between baseline and filtering tasks (i.e., Garner interference) than participants assigned to discrimination training, in RT, *F*(1,33) = 4.30, *p* = 0.05, MS_e_ = 386.58, η^2^= 0.005, but not in accuracy, *F*(1,33) = 0.04, ns, MS_e_ = 0.003, η^2^< 0.001. Across groups, Garner interference was significantly smaller after training in both RT, *F*(1,33) = 21.08, *p* < 0.001, MS_e_ = 266.92, η^2^= 0.02, and in performance accuracy, *F*(1,33) = 9.07 *p* < 0.01, MS_e_ = 0.001, η^2^= 0.02, suggesting an experience-dependent improvement in selective attention. The type of training participants underwent determined the degree of their improvement in selective attention: As shown in **Figure 3**, participants assigned to inhibition training showed a significantly greater reduction from pretest to posttest in Garner interference than participants assigned to discrimination training, creating a Train × Task × Test interaction in both RT, *F*(1,33) = 6.85, *p* = 0.01, MS_e_ = 266.92, η^2^= 0.005, and (marginally) in accuracy, *F*(1,33) = 3.46, *p* = 0.07, MS_e_ = 0.001, η^2^= 0.01. Moreover, the beneficial effects of inhibition training on Garner interference in RT were largely restricted to the poor attenders at pretest, resulting in a significant Skill × Train × Test × Task interaction (see **Figure 4**), *F*(1,33) = 6.24, *p* = 0.02, MS_e_ = 266.92, η^2^= 0.005, but not in accuracy, *F*(1,33) < 0.001, ns, MS_e_ = 0.001, η^2^< 0.001.

**Table 1:** Reaction times and accuracies, with accompanying standard errors (SE), in baseline and filtering (separately for congruent, neutral, and incongruent trials) tasks at pretest and posttest for participants in discrimination and inhibition training groups.

Task	Pretest				Posttest			
	*RT*	*SE*	*Accuracy*	*SE*	*RT*	*SE*	*Accuracy*	*SE*
**Discrimination training**								
Baseline	455	11.88	95	1.26	420	9.73	97	0.58
Congruent	414	14.63	98	0.48	386	11.41	98	0.40
Neutral	491	14.77	95	1.33	441	10.66	96	0.60
Incongruent	610	30.51	76	3.39	555	28.74	85	2.76
**Inhibition training**								
Baseline	474	14.80	97	0.67	444	13.09	97	0.59
Congruent	445	19.76	98	0.48	417	13.38	98	0.41
Neutral	514	20.11	96	0.58	445	12.50	97	0.57
Incongruent	713	45.53	66	4.14	587	38.14	79	4.50

**FIGURE 5:**
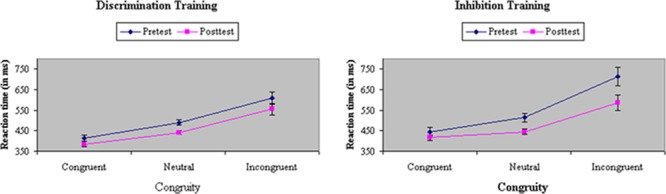
Reaction time (in ms) on congruent, neutral, and incongruent trials in the filtering task at pretest and posttest in discrimination and inhibition training groups. Inhibition training was effective in reducing distraction from incongruent flankers, yielding a significant Train × Congruity × Test interaction.

Analyses of variance of RTs in filtering tasks showed a main effect of Congruity in both RTs, *F*(2,66) = 171.31, *p* < 0.001, MS_e_ = 4413.64, η^2^= 0.58, and accuracy, *F*(2,66) = 182.94, *p* < 0.001, MS_e_ = 0.03, η^2^= 0.85. Participants responded fastest and most accurately on congruent trials (415 ms, 98%), slowest and least accurately on incongruent trials (612 ms, 77%), and were intermediate on neutral trials (471 ms, 96%). Participants assigned to inhibition training showed a relatively greater difference before training in performance between congruent and incongruent trials (i.e., flanker congruity effect) in RT, *F*(2,66) = 4.18, *p* = 0.01, MS_e_ = 2818.71, η^2^= 0.01, but not in accuracy, *F*(2,66) = 0.39, ns, MS_e_ = 0.02, η^2^= 0.001). The Stroop congruity effect was reduced after training in both RTs, *F*(2,66) = 12.16, *p* < 0.001, MS_e_ = 1385.48, η^2^= 0.01, and accuracy, *F*(2,66) = 17.94, *p* < 0.001, MS_e_ = 0.01, η^2^= 0.03. Inhibition training was effective in reducing distraction from incongruent flankers, which, as shown in **Figure 5**, led to a significant Train × Congruity × Test interaction in both RTs, *F*(2,66) = 4.35, *p* = 0.02, MS_e_ = 1385.48, η^2^= 0.005, and in accuracy, *F*(2,66) = 4.64, *p* = 0.01, MS_e_ = 0.01, η^2^= 0.01.

**FIGURE 6:**
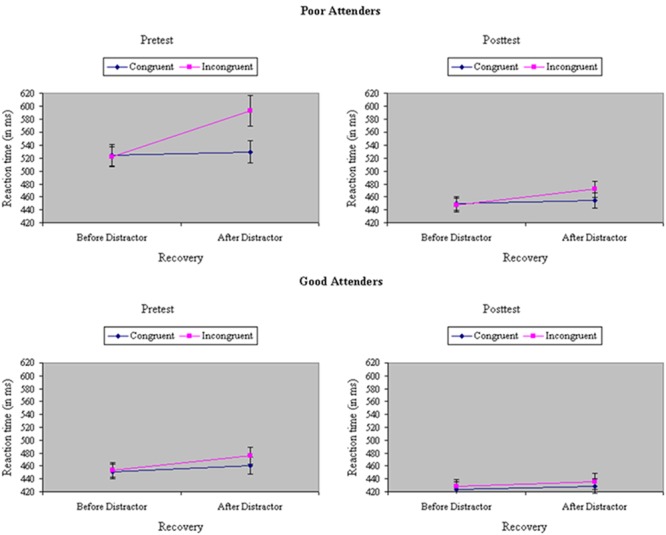
Reaction time (in ms) on neutral trials in the filtering task, before versus after a trial with a congruent or incongruent flanker (distraction recovery), at pretest and posttest, separately by poor attenders (Top) and good attenders (Bottom). The effect of training on recovery was especially prominent in RTs after incongruent trials, an outcome restricted to participants showing relatively poor selective attention at pretest, yielding a significant Skill × Recovery × Test × Congruity interaction.

Analyses of variance were performed on RTs and accuracies during filtering trials with neutrals flankers, before versus after a trial with a congruent or incongruent flanker, a variable termed distraction recovery. Participants were relatively slower, *F*(1,33) = 75.12, *p* < 0.001, MS_e_ = 342.77, η^2^= 0.04, and less accurate, *F*(1,33) = 5.07, *p* < 0.05, MS_e_ = 0.01, η^2^= 0.05, to respond to neutral trials after either a congruent or an incongruent trial, indicative of the carryover of distraction. The carryover effects were particularly severe after incongruent trials, creating a Recovery × Congruity interaction, at least in RTs, *F*(1,33) = 40.68, *p* < 0.001, MS_e_ = 280.99, η^2^= 0.02 [accuracy: *F*(1,33) = 0.10, ns, MS_e_ = 0.004, η^2^< 0.001]. However, training reduced the difference in speed, *F*(1,33) = 23.69, *p* < 0.001, MS_e_ = 200.21, η^2^= 0.01, but not accuracy, *F*(1,33) = 2.65, ns, MS_e_ = 0.01, η^2^= 0.02, to neutral trials before versus after congruent or incongruent trials, suggesting experience-dependent improvement in the recovery from distraction. The effect of training on recovery was especially prominent in RTs after incongruent trials (Recovery × Test × Congruity), *F*(1,33) = 16.52, *p* < 0.001, MS_e_ = 211.14, η^2^= 0.01 [accuracy: *F*(1,33) = 1.16, ns, MS_e_ = 0.01, η^2^= 0.01], an outcome restricted to participants showing relatively poor selective attention at pretest (Skill × Recovery × Test × Congruity), *F*(1,33) = 6.60, *p* = 0.01, MS_e_ = 211.14, η^2^= 0.002, as shown in **Figure 6** [accuracy: *F*(1,33) = 3.69, *p* < 0.05 MS_e_ = 0.01, η^2^= 0.04].

### ERP Effects: Target

**FIGURE 7:**
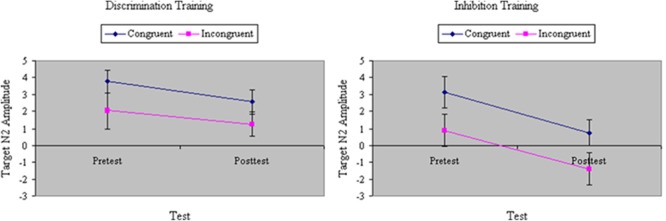
Amplitude of N2 event-related potential (ERP) component to targets (in microvolts) on congruent and incongruent trials in the filtering task at pretest and posttest in discrimination and inhibition training groups. Enhancement from training in N2 amplitude was equivalent for congruent and incongruent stimuli.

The difference at pretest in RTs between congruent and incongruent stimuli was used as a covariate in all ERP analyses. Notably, the results reported here were unaffected by the pretest RT congruity effect. As hypothesized, we found a main effect of Congruity in N2 peak amplitude to the target, *F*(1,32) = 20.16, *p* < 0.001, MS_e_ = 57.98, η^2^= 0.22; N2 amplitude was significantly greater on incongruent than congruent trials. Training increased the amplitude of N2, *F*(1,32) = 27.29, *p* < 0.001, MS_e_ = 35.84, η^2^= 0.18, particularly for participants undergoing inhibition training, *F*(1,32) = 5.18, *p* < 0.05, MS_e_ = 35.84, η^2^= 0.03, and especially for those in that group with poor attention skill, *F*(1,32) = 6.97, *p* < 0.05, MS_e_ = 35.84, η^2^= 0.05. However, in each group we found that the enhancement from training in N2 amplitude was equivalent for congruent and incongruent stimuli (see **Figure 7**), *F*(1,32) = 0.10, ns, MS_e_ = 14.59, η^2^= 0.0002. Hence, neither inhibition training nor discrimination training affected the magnitude of the N2 congruity effect to target stimuli.

**FIGURE 8:**
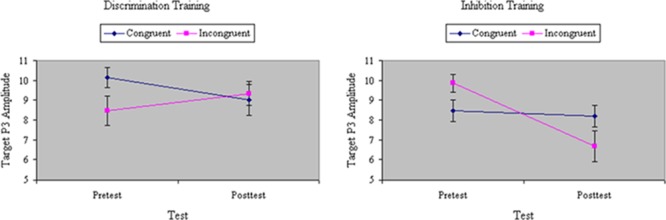
Amplitude of P3 ERP component to targets (in microvolts) on congruent and incongruent trials in the filtering task at pretest and posttest in discrimination and inhibition training groups. Whereas inhibition training yielded a significant decrease in the amplitude of P3 to incongruent targets, discrimination training slightly boosted P3 to these stimuli, yielding a significant three-way interaction of Training, Group, and Congruity.

Analyses of variance of target P3 amplitude revealed a main effect of Training, *F*(1,32) = 6.86, *p* < 0.05, MS_e_ = 46.62, η^2^= 0.12, with the magnitude of P3 decreasing from pretest to posttest. However, the effect of training was largely restricted to the inhibition group, *F*(1,32) = 5.10, *p* < 0.05, MS_e_ = 46.62, η^2^= 0.09. There was no effect of Congruity, *F*(1,32) = 1.11, ns, MS_e_ = 53.20, η^2^= 0.02. Yet there was a significant three-way interaction of Training, Group, and Congruity, *F*(1,32) = 15.64, *p* < 0.001, MS_e_ = 33.12, η^2^= 0.19. As can be seen in **Figure 8**, whereas inhibition training yielded a significant decrease in the amplitude of P3 to incongruent targets, discrimination training slightly boosted P3 to these stimuli.

### ERP Effects: Distractor

**FIGURE 9:**
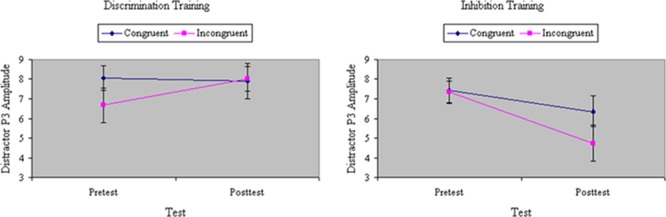
Amplitude of P3 ERP component to distractors (in microvolts) on congruent and incongruent trials in the filtering task at pretest and posttest in discrimination and inhibition training groups. The distinct effects of inhibition or discrimination training on distractor P3 were mainly relegated to incongruent stimuli, yielding a significant three-way interaction of Training, Group, and Congruity.

**FIGURE 10:**
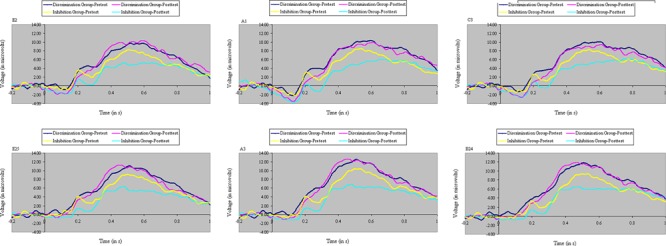
Event-related potential waveforms to the initial flanker on incongruent trials at pretest and posttest over six centroparietal electrode locations (E2, A1, C3, E25, A3, and B24), separately for participants in discrimination training and inhibition training. Inhibition training decreased P3 to these stimuli, 400–900 ms after stimulus onset, whereas discrimination training increased P3 to these stimuli.

Analyses of variance of P3 amplitude to the initial flanker revealed a marginal main effect of Congruity, *F*(1,32) = 3.58, *p* < 0.07, MS_e_ = 59.69, η^2^= 0.07, with relatively weaker P3 magnitude to incongruent stimuli. The congruity effect in P3 was equal between groups before training, *F*(1,32) = 1.64, ns, MS_e_ = 39.78, η^2^= 0.02. There was no main effect of Training, *F*(1,32) = 2.64, ns, MS_e_ = 52.98, η^2^= 0.04, but there was a significant interaction between Training and Group, *F*(1,32) = 9.58, *p* < 0.01, MS_e_ = 52.98, η^2^= 0.16: Inhibition training decreased, whereas discrimination training increased, P3 amplitude from pretest to posttest. As shown in **Figure 9**, the distinct effects of inhibition or discrimination training on distractor P3 were mainly relegated to incongruent stimuli, yielding a significant three-way interaction of Training, Group, and Congruity, *F*(1,32) = 5.69, *p* < 0.05, MS_e_ = 36.68, η^2^= 0.06. **Figure 10** depicts ERP waveforms to incongruent stimuli over centroparietal sites at pretest and posttest in each of the two training groups.

**FIGURE 11:**
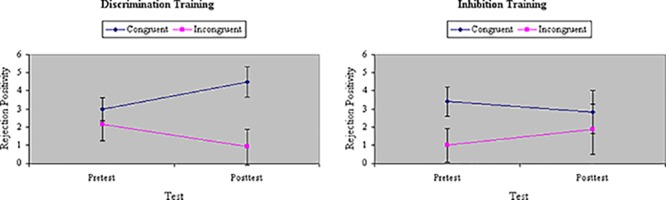
Amplitude of RP ERP component to distractors (in microvolts) on congruent and incongruent trials in the filtering task at pretest and posttest in discrimination and inhibition training groups. Inhibition training increased RP to incongruent stimuli whereas discrimination training significantly decreased RP to incongruent stimuli.

Analyses of variance of RP to the initial distractor showed a main effect of Congruity, *F*(1,32) = 17.19, *p* < 0.001, MS_e_ = 56.85, η^2^= 0.21, with RP magnitude significantly greater to congruent than incongruent stimuli. The difference in RP between congruent and incongruent stimuli was comparable between groups at pretest, *F*(1,32) = 1.60, ns, MS_e_ = 46.00, η^2^= 0.02. There was no main effect of Training, *F*(1,32) = 0.19, ns, MS_e_ = 90.84, η^2^= 0.004, and no interaction between Training and Group, *F*(1,32) = 0.04, ns, MS_e_ = 90.84, η^2^= 0.001. However, there was a significant three-way interaction among Congruity, Training, and Group, *F*(1,32) = 4.87, *p* < 0.05, MS_e_ = 52.23, η^2^= 0.06. As **Figure 11** shows, inhibition training numerically increased RP to incongruent stimuli whereas discrimination training decreased RP to incongruent stimuli, though only the latter effect was statistically significant in planned comparisons.

### Source Analysis

**FIGURE 12:**
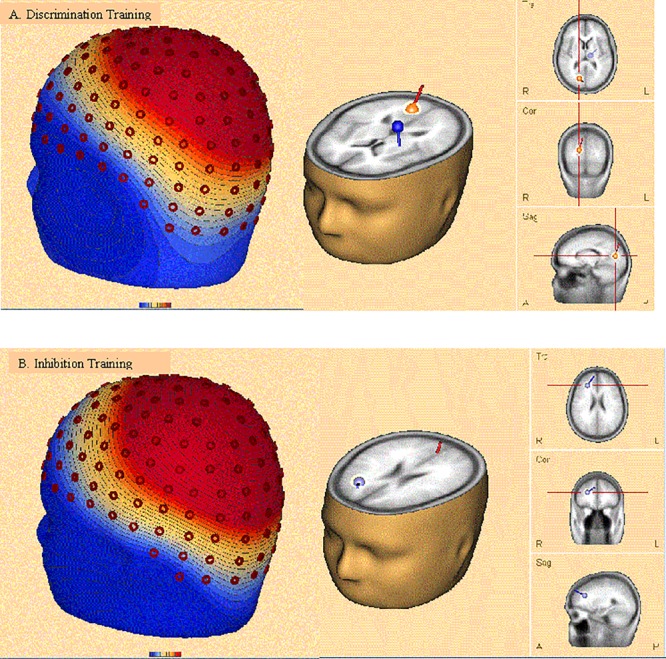
Topographic map (Leftmost image in each panel) and discrete dipole source analysis solution (Rightmost image) performed separately for discrimination training **(A)** and inhibition training **(B)** at posttest to incongruent distractors during an epoch 600–700 ms after stimulus onset, corresponding to the RP and P3 ERP components. After discrimination training, the best fitting dipoles were located in the right occipital lobe and the cingulate gyrus. After inhibition training, the best fitting dipoles were located in the right occipital lobe and the medial frontal gyrus.

Discrete dipole source analysis was performed separately for each type of training at posttest to incongruent distractors during an epoch 600–700 ms after stimulus onset, corresponding to the RP and P3 ERP components. As shown in Panel A of **Figure 12**, after discrimination training two dipoles provided the best fit across 160 electrode sites (residual variance [RV] = 5.2%), the first in the right occipital lobe (Brodman area 17; Talairach coordinates: *x* = 15.0, *y* = -73.9, *z* = 9.7) and the second in the left limbic lobe (cingulate gyrus; Talairach coordinates: *x* = -12.3, *y* = -17.8, *z* = 25.1). After inhibition training, again two dipoles provided the best fit (RV = 5.4%), as depicted in Panel B of **Figure 12**. The first dipole was again located in the right occipital lobe (Brodman area 18; Talairach coordinates: *x* = 3.3, *y* = -67.7, *z* = 4.6). However, the second dipole was located in the medial frontal gyrus (Talairach coordinates: *x* = 25.5, *y* = 32.8, *z* = 23.6).

### Correlational Analyses

**FIGURE 13:**
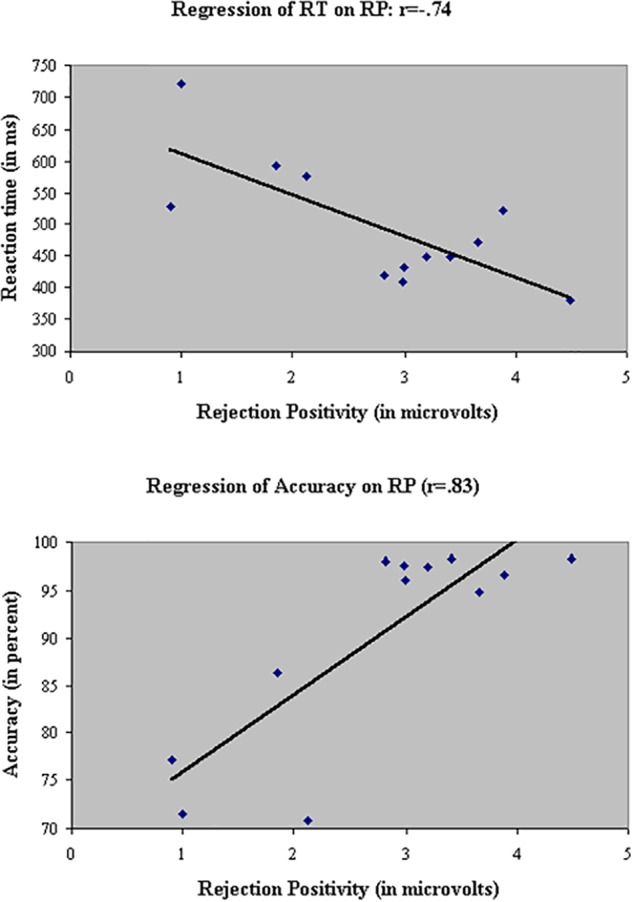
Linear regressions of the two behavioral measures, RT (Top) and accuracy (Bottom), on RP to the initial distractor. As RP increased, participants responded faster (*r* = –0.74) and more accurately (*r* = 0.83).

Linear regressions were performed across groups, test sessions, and stimulus types in the filtering task to probe the relationship between RP and the two behavioral measures, RT (**Figure 13**, top panel) and accuracy (**Figure 13**, Bottom panel). RP was significantly associated with both measures: As RP to the initial distractor increased, participants responded faster (*r* = -0.74) and more accurately (*r* = 0.83).

## Discussion

Two groups of healthy young adults were exposed to 3 weeks of cognitive training in a modified version of the visual flanker task, one group trained to focus on the target (discrimination training) and the other group to ignore the flankers (inhibition training). Inhibition training, but not discrimination training, led to a significant reduction in Garner interference, indicating improved selective attention, and a significant reduction in Stroop interference, indicating more efficient resolution of stimulus conflict. The behavioral gains from training were greatest in participants who showed the poorest selective attention at pretest.

Electrophysiological recordings revealed that inhibition training decreased the magnitude of the P3 ERP component in both the target and the distractor when the stimulus pairing was incongruent. Moreover, inhibition training increased the magnitude of RP to incongruent distractors, suggesting an improvement in inhibitory control. Source modeling of incongruent distractors in the RP time epoch uncovered a dipole in the medial frontal gyrus for participants receiving inhibition training, but in the cingulate gyrus for participants receiving discrimination training.

### Contribution to Previous Research

Chen and Melara (2014) developed a formal computational model (tectonic theory; see also Melara and Algom, 2003) that precisely predicted behavioral outcomes (trial-by-trial RTs) from individual distractor RP amplitudes, revealing a strong link between pre-target inhibition and target selection and decision making processes. Melara et al. (2002) found that by enhancing distractor RP inhibition training was effective in improving auditory selective attention. In the current study we observed that a form of inhibition training applied to a visual conflict task created experience-dependent elevations in distractor RP specific to incongruent target-flanker pairings. Correlational analyses indicated that the changes in inhibitory processing, as measured by RP, were strongly related to behavioral performance in the flanker task, for both RT and accuracy. Our use of a separate discrimination training group revealed that the experience-dependent changes we found were specific to inhibition training. Thus, the current study extends previous research by demonstrating that inhibitory processing during selective attention is malleable in both the auditory and visual modalities, and that improved inhibitory processing is associated with better attention performance in both conflict and nonconflict situations.

Millner et al. (2012) found that practice on the Simon task reduced the difference in N2 amplitude on the flanker task between congruent and incongruent trials. Our results differed in showing no effect of either discrimination or inhibition training on the N2 congruity effect in the flanker task. The current results pointed instead to the role of inhibition training in suppressing P3 amplitude on conflict trials during both target and distractor processing. In our version of inhibition training the signal-to-noise ratio was progressively decreased across repeated blocks of trials. We speculate here that such training effectively weakened distractor salience (i.e., P3 effect), while leaving undisturbed the initial registration of conflict on incongruent trials (i.e., no N2 effect). If N2 amplitude on conflict trials is a measure of conflict monitoring (Van Veen and Carter, 2002), then our results suggest that the inhibition training used here does not habituate the conflict signal, a possible difference with the training procedure used by Millner et al. (2012), which involved the repeated presentation of equally salient conflict trials. Their study did not include a control group nor was it possible for them to take separate neural measures of target and distractor processing. The results of our study extend those of Millner et al. (2012) by demonstrating that, relative to discrimination training, the neural effects of inhibition training in the flanker task (i.e., P3 and RP) are specific to incongruent distractors.

### How Does Inhibition Training Affect Conflict Resolution?

Previous research (Melara et al., 2002, 2012) led us to expect that inhibition training would weaken the perceived salience of distractors across all trial types, as measured by the P3 component. Yet the current results show that the effects of inhibition training apply only to incongruent trials, and extend across both target and distractor processing: At posttest, P3 amplitude on incongruent trials was diminished from pretest levels for both targets and distractors. This finding, coupled with the absence of an N2 training effect, suggests that inhibition training acts to lessen the perceived salience of all stimuli held in working memory during moments of stimulus conflict. On this view, the improved speed and accuracy of performance we observed at posttest resulted from training-based suppression of the conflict signal whenever the target and flanker mismatched. As the awareness of target-flanker conflict lessens (from repeated practice at suppression), response competition becomes less severe, and hence less disruptive to performance. Thus, perhaps inhibition training does less to speed conflict resolution than to diminish the deleterious impact on performance of conflict awareness in working memory. Suppression of conflict in working memory may also explain changes from training in the carryover of distraction: Participants undergoing inhibition training demonstrated better recovery from conflict on subsequent neutral trials. One reason may be that the awareness of conflict dissipated from memory more quickly in these participants, relative both to their pretest levels and to those practiced in discrimination, and so was less disruptive on subsequent trials.

### Implications for Individual Differences in Distractibility

Inhibition training was especially effective at reducing interference and speeding recovery in those participants who showed the highest levels of distractibility at pretest. If inhibition training indeed mitigates the salience of conflict in working memory, the current results suggest that participants with poor attention skill benefit most from learning to control working memory. Although unmeasured in the present study, previous research has found that larger working memory capacity predicts superior performance in tasks of selective attention (Engle et al., 1999) because those with large capacity are better able to suppress task-irrelevant information (Gulbinaite et al., 2014). This may be why improvements in performance after attention training are often greatest for participants with poor executive functioning (Diamond and Lee, 2011; Millner et al., 2012). In this regard, the plasticity in inhibitory control suggested by our results has implications for treating psychiatric syndromes involving compromise to executive functions of working memory, including individuals with Post-Traumatic Stress Disorder (Clark et al., 2003; Leskin and White, 2007; Aupperle et al., 2012; Harrington et al., 2012), Attention Deficit Hyperactivity Disorder (Nigg, 2001), substance use disorder (Jentsch and Taylor, 1999), and personality disorders (Depue and Lenzenweger, 2001; Fertuck et al., 2006).

### Implications for Conflict Adaptation

Attention theories focus on the flexible and adaptive nature of cognition when humans are confronted with environmental conflict (Gratton et al., 1988, 1992; De Jong et al., 1994; Botvinick et al., 1999, 2004). According to the conflict adaptation model (Botvinick et al., 2001; Kerns et al., 2004), control adjustments imposed by prefrontal neural mechanisms resolve stimulus conflict when performing attention tasks. On this account, top-down attentional control divides into two functions: conflict monitoring, handled physiologically by the anterior cingulate cortex (ACC), and implementation, the purview of the PFC (Botvinick et al., 2001). When participants perform the flanker task, for example, the ACC detects conflict whenever a flanker (e.g., vertical orientation) mismatches a target (e.g., horizontal orientation). Neural activity subsequently directed from ACC to PFC signals the need for enhanced attentional control, which is then implemented to relieve interference and ensure efficient task performance.

In the current study we found dipole source activity in PFC on conflict trials after inhibition training relative to participants undergoing discrimination training. Inhibition training may have enhanced control adjustment in the flanker task by elevating the basal activation of executive control centers in the frontal cortex, a conclusion in keeping with the conflict adaptation model (Botvinick et al., 2001; Kerns et al., 2004). Enhanced PFC activity from greater control adjustment after incongruent trials (i.e., conflict adaptation) may also explain the enhanced distractor recovery on subsequent trials that resulted from inhibition training (Botvinick et al., 1999; Durston et al., 2003).

### Limitations

The current study revealed little change in performance to targets or distractors after discrimination training. Yet previous research using discrimination training has shown robust change in both behavioral and electrophysiological indices (e.g., Tremblay et al., 1997; Atienza et al., 2002), even after a single training session (Wright and Sabin, 2007). One possibility is that in providing repeated practice in distinguishing the identical target pairs our training paradigm proved ineffective in enhancing perceptual discrimination. An alternative approach would be to introduce progressively finer visual discriminations over the course of practice, a procedure that has been effective in other training studies (Tong et al., 2009).

Our analysis of individual differences was restricted to the effects of premorbid attentional skill on attentional learning. Yet previous research has shown that individual differences in working memory capacity predict selective attention success (Engle et al., 1999; Long and Prat, 2002; Heitz and Engle, 2007). It is conceivable that memory capacity also predicts attentional learning. For example, perhaps individuals with high working memory capacity, who already show relatively good inhibitory control (Gulbinaite et al., 2014), benefit less from inhibitory training than individuals with low working memory capacity. Future research could fruitfully probe how individual differences in working memory affect the plasticity of attention.

## Conclusion

In the current study we found that executive control improves after training to suppress incongruent distractor stimuli, but not after training involving practice at target discrimination. Greater executive control from inhibition training was evident in both better selective attention (decreased Garner interference) and more efficient resolution of conflict (decreased Stroop interference), with neural correlates observed in the Rejection Positivity ERP component and a complementary neural source in the PFC. The present study extends findings on inhibition training to the realm of conflict resolution, demonstrating that inhibitory processes are plastic, particularly in individuals with poor attention skills.

## Ethics Statement

This study was carried out in accordance with the recommendations of Institutional Review Board of The City University of New York with written informed consent from all subjects. All subjects gave written informed consent in accordance with the Declaration of Helsinki. The protocol was approved by the Institutional Review Board of The City University of New York.

## Author Contributions

RM contributed to three domains of the research project: (1) Research Design, including conception and analysis and interpretation of data; (2) Research Reporting, including drafting of the manuscript and critical revision of the manuscript for important intellectual content; and (3) Research Support, including statistical analysis, obtaining funding, administrative and technical support, and supervision. SS contributed to three domains of the research project: (1) Research Design, including conception; (2) Research Reporting, including critical revision of the manuscript for important intellectual content; and (3) Research Support, including obtaining funding, technical support, and supervision. DH contributed to three domains of the research project: (1) Research Design, including interpretation of data; (2) Research Reporting, including critical revision of the manuscript for important intellectual content; and (3) Research Support, including obtaining funding and supervision.

## Conflict of Interest Statement

The authors declare that the research was conducted in the absence of any commercial or financial relationships that could be construed as a potential conflict of interest.
